# Simple method for the quantitative analysis of thin copolymer films on substrates by infrared spectroscopy using direct calibration

**DOI:** 10.1039/c7ay01748k

**Published:** 2017-08-21

**Authors:** Martin Tazreiter, Paul Christian, Robert Schennach, Thomas Grießer, Anna Maria Coclite

**Affiliations:** a Institute of Solid State Physics , NAWI Graz , Graz University of Technology , 8010 Graz , Austria . Email: anna.coclite@tugraz.at; b Department Kunststofftechnik , Montanuniversität Leoben , 8700 Leoben , Austria

## Abstract

The chemical composition of a copolymer drives many important material properties and quantification in terms of comonomer volume fraction is thus of practical relevance for many studies. Infrared spectroscopy is one of the most common techniques for compositional analysis but it usually relies on manual evaluation of baselines and peak heights, which can be rather inaccurate and become a laborious task when having multiple spectra to evaluate. On the contrary, Maxwell's theory of electrodynamics can be used to calculate the complex index of refraction from measured spectra promising a more accurate quantification. Since this procedure is rather involved, we propose a simple in-house developed IR-quantification routine to automatically evaluate the comonomer volume fractions of thin copolymer films by using the Bouguer–Lambert–Beer approximation after correcting the baseline of all absorbance spectra automatically. This method was experimentally evaluated on over 40 thin polymeric coatings synthesized by initiated chemical vapor deposition on silicon substrates. The samples comprised a wide range of different compositions and were synthesized from four different monomers, with single films consisting of up to three components. All data obtained by our routine was compared with data from spectroscopic ellipsometry and with X-ray photoelectron spectroscopy data of selected samples. The comparisons show that the IR-quantification routine reliably evaluated the polymer composition even when the involved comonomers exhibited similar chemistry, as it is the case for methacrylic acid cross-linked with ethylene glycol dimethacrylate.

## Abstract 2

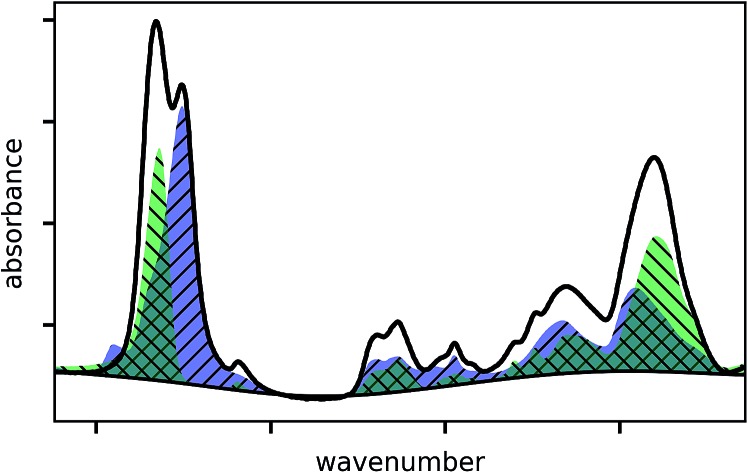
Automated baseline estimation followed by least squares fitting of copolymer spectrum allows quantification in terms of comonomer volume fraction.

## Introduction

Thin polymer coatings, below 1 μm, are an attractive technology for physical and chemical modification of a wide variety of different surfaces, being employed in both industrial but also research-based applications. Such coatings ensure biocompatibility for implants or medical instruments,^1^ are applied to devices as protective coatings against thermal,^2^ chemical^3^,^4^ or physical influences^5^ but are also utilized in large-scale applications such as the fabrication of vehicle tires, where they are used as mould release agents.^6^ That polymeric coatings can be used in such a plethora of different applications stems largely from the wide range of chemistries accessible within this class of materials, which can be further tuned by the inclusion of inorganic materials such as nanoparticles.^7^ Copolymers are a particularly interesting class of materials in this regard, combining the individual properties of two or more constituents into a single polymer. This allows, for example, to combine hydrophilic monomers such as (hydroxyethyl)methacrylate (HEMA) with cross-linkers like ethylene glycol dimethacrylate (EGDMA), thus forming a stable hydrogel when immersed into water.^8^ Among properties such as average molecular weight, polydispersity or aggregation state (*e.g.* amorphous, liquid-crystalline), the relative fraction of the constituent monomers in the resulting polymer is often a defining characteristic. For example, the maximal water-uptake in p-(HEMA-*co*-EGDMA) exhibits a linear dependence on the HEMA content, until stability cannot be maintained by the decreasing EGDMA fraction.^9^ Exact data on the volume fractions are also crucial inputs to several analysis methods such as comonomer reactivity evaluation by the Fineman–Ross method^10^ or the characterization of charge transport properties in ion-containing copolymers^11^ and are thus of practical importance.

While for soluble polymers a profusion of chemical analysis is available (mass spectrometry, chromatography, calorimetry) for cross-linked or fluorinated polymers the precise characterization of the chemical composition is more complicated. A standard technique to evaluate the relative composition of thin copolymer films is Fourier transform infrared spectroscopy (FTIR).^12^ FTIR is a relatively easy and fast technique when compared to mass spectrometry, titration or chromatography and it is available in most laboratories. In some cases, the chemical bonds in the copolymer produce distinguishable characteristic peak positions in the absorbance spectra, due to their respective chemical environments (*e.g.* a C=O bond in a ketone and in carboxylic acid absorb at different wavenumbers). The respective volume fraction of the involved monomers may then be evaluated by baseline correcting the absorbance spectrum and comparing the different peak heights or areas utilizing the Bouguer–Lambert–Beer^13^ relation or a weakened relation in form of a calibration curve. This procedure is commonly used in literature, but the exact method of how the baseline is corrected is often not stated, which is problematic since this can influence the outcome. Usually one-point and two-points corrections are applied, which subtract a constant and a straight line from the absorbance spectrum, respectively. In the absence of interference fringes, this can be sufficient. In the presence of interference fringes, splines can be used with manually defined points in locations, which the user interprets to be the baseline. This obviously produces user dependent outcomes which can vary from spectrum to spectrum. The method presented in this paper uses an automated baseline correction to reduce these problems. For quantification purposes, the method of how the baseline corrected absorbance spectrum is used further also varies a lot within literature. Sometimes absorbance values at specific wavenumbers are used^14^–^17^ and other times areas under peaks are used.^18^–^21^ When the absorbance values at specific wavenumbers are used, more noise than necessary is extracted from the spectrum. Using the whole area of a peak reduces the noise. Both only work in case of non-overlapping peaks. When peaks overlap, they might be fitted with multiple Gauss or Lorentz functions (or a convolution of both), but the necessary number of fitted peaks as well as their type and constrains on their parameters introduces again a user influence on the outcome. In the present study, these problems are minimized by using homopolymer spectra themselves to fit copolymer spectra. Some studies use an alternative approach called principal component analysis.^22^,^23^ Theoretically more significant than the partly experimental problems stated above is that the presence of electric field standing wave effects as, *e.g.* interference fringes in the spectrum, cannot be directly described by the Bouguer–Lambert–Beer relation which is therefore an approximation.^24^,^25^

The theory which accurately describes both absorption and electric field standing wave effects in transmission spectra is Maxwell's theory of electrodynamics.^26^–^28^ The central quantity which characterizes the properties of waves in or at the interface of materials is the complex index of refraction, which is wavenumber dependent. The Kramers–Kronig relation relates the real part to the imaginary part and *vice versa*, but requires the knowledge of one part at all frequencies.^29^ It is straightforward to calculate the transmission function of a free-standing layer at normal incidence with perfectly smooth surfaces given the complex and wavenumber dependent index of refraction and the thickness. The inverse problem to calculate the index of refraction from one measured transmission spectrum is naturally more involved but still possible. R. T. Graf *et al.*^30^ for example determined the complex index of refraction of free standing organic films from their transmission spectra, by using the interference fringes to determine the film thickness and index of refraction at very high frequency *n*_∞_ for subsequent calculation of the wavenumber dependent complex index of refraction. R. Uitz *et al.*^31^ used transmission and reflection spectra of thin polymer films to determine the index of refraction. With this method, care must be taken since it might yield multiple solutions.^32^

These methods get more complicated if during the transmission measurement a substrate is present in addition to the investigated layer. Nilsson^33^ measured transmission at normal incidence through a layer on a transparent substrate and showed how the optical constants can be calculated. In case of an absorbing substrate like silicon the formulas would have to be adapted. If the layer is thin such that only a fraction of the interference fringe period is visible in the spectrum, the thickness and the refractive index *n*_∞_ cannot be reliably determined from the interference pattern. Unknown surface roughnesses can further complicate the procedure. The Maxwell compatible methods discussed so far are model independent and give the index of refraction at discretized wavenumbers as output. An also Maxwell compatible method, which is model dependent, is Dispersion Analysis^34^,^35^ which uses a model for the dielectric function, often a sum of Drude–Lorentz oscillators. In the standard approach the number of oscillators and therefore the degrees of freedom are kept as small as possible but as large as to get a good fit to the data. A. B. Kuzmenko^36^ on the other hand used a number of oscillators on the order of the number of discretized wavenumbers and therefore extended it to an essentially model independent method. Even though Dispersion Analysis is a useful tool, it does not remove the partly experimental problems stated before. Since for this work a simple quantification method was desired which does not depend on preknowledge of layer thickness or refractive index (except for the homopolymer spectra), the Bouguer–Lambert–Beer approximation was utilized in combination with an automatic baseline correction algorithm to remove interference fringes. Other symptoms of the electric field standing wave effect like altered band ratios and band positions were neglected. The validation of the obtained values will justify that. The polymer layers used in this work were synthesized by initiated Chemical Vapor Deposition (iCVD). iCVD is a versatile method to fabricate thin polymer coatings on a variety of different surfaces,^37^ which has been successfully used in the preparation of hydrophobic surfaces for dropwise condensation,^38^ as antifouling^39^ and anticorrosion^40^ coatings or for drug encapsulation.^41^,^42^ The iCVD method is a solvent-free polymerization method, where all the reactants are supplied from the vapour phase. The process relies on a free radical polymerization mechanism and allows obtaining polymers and copolymers with high chemical fidelity, contrarily to other vapour deposition techniques. In general, this method results in coating thicknesses ranging from only a few nanometres to some micrometres. The commonly used monomers are acrylates, which often exhibit similar chemistry. Films deposited by this technique can be used as test models for the evaluation of the volume fraction analysis due to their high chemical structure retention, lack of unpredictable side-reactions and low inclusion of initiator radicals in the initiation and termination phase.

The present work shows an approach to evaluate the composition of such films by treating the experimental FTIR absorbance spectra of the copolymers as a combination of the respective homopolymer spectra. The aim is to provide an easy and reliable tool for copolymer analysis, which eliminates the person-to-person variability. The IR-quantification method was evaluated on experimental data for different thin polymer films deposited by iCVD and was shown to work also for copolymers including three different comonomers. The IR-quantification routine and a graphical user interface were implemented using the Python programming language. The scripts are available online free of charge https://www.annacoclite.com/equipment/.

## Theory

**Fig. 1:**
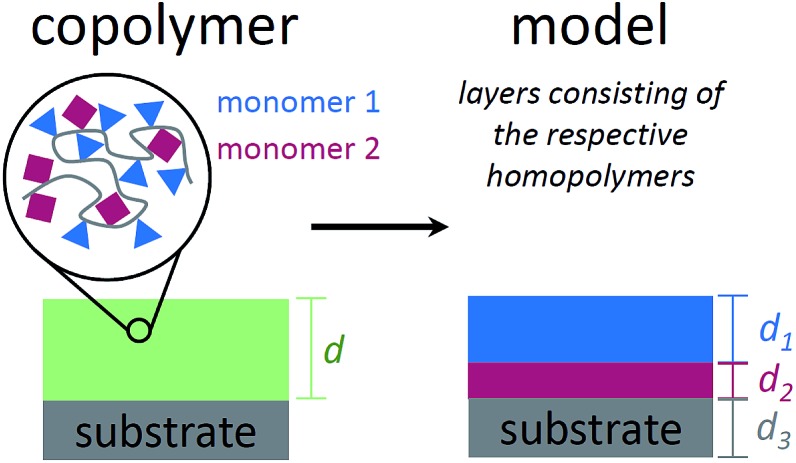
Schematic representation of a copolymer film on a substrate (left) and the model system used to describe the infrared absorbance of such a system (right). The polymer film is treated as a stack of homopolymer layers (one for each comonomer), with the layer thicknesses corresponding to the respective volume fractions.

To describe the absorbance of an arbitrary copolymer film of thickness *d*, a model system is employed. The film is treated as a layered system of independent homopolymer films, each layer corresponding to one of the comonomers (see Fig. 1 for a schematic representation).

The individual layer thickness is then proportional to the respective comonomer volume fraction. As will be shown, the absorbance calculated from this model is equal to the copolymer's absorbance within the Bouguer–Lambert–Beer^13^ approximation (given that some prerequisites are met, which are stated at the end of this section). According to the Bouguer–Lambert–Beer approximation, the decrease of radiant power for transmission through *N* + 1 layers of different materials with absorbance per unit length *A*_*i*_ and thicknesses *d*_*i*_, can be expressed as:
1

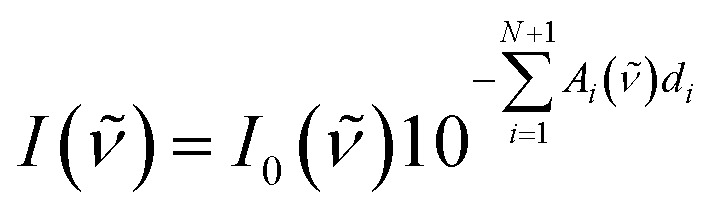


*I*_0_(*ν̃*) is the radiant power of the incoming light and *I*(*ν̃*) is the radiant power of the outgoing light, which is measured indirectly by the FTIR technique. Each material *i* for *i* = 1…*N* describes a (homo-)polymerized version of one of the monomers. The (*N* + 1)^th^ layer denotes the substrate (bulk silicon in the present case), which can be eliminated from the equation by a reference measurement of the pristine substrate:

2 *I*_sub_(*ν̃*) = *I*_0_(*ν̃*)10^–*A*_*N*+1_(*ν̃*)*d*_*N*+1_^

By dividing eqn (1) by (2), one eliminates the substrate influence (which in case of Si is actually for large parts due to reflectance) as well as *I*_0_(*ν̃*), which is depending on the infrared source:
3

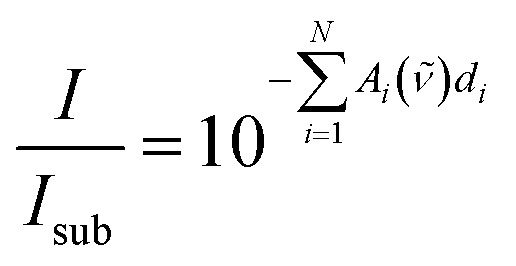




Taking the base-10 logarithm and multiplying by –1, one obtains the copolymer absorbance spectrum *A*(*ν̃*)
4

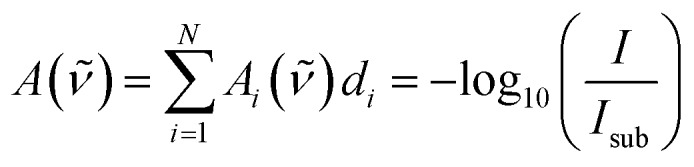




It was assumed that all *N* polymer species are separated into layers of thickness *d*_*i*_, but as evident from eqn (1), the measured *I*(*ν̃*) would not change if the layers are (physically) intermixed to an arbitrary concentration dependence perpendicular to the surface. This holds under the assumption that the total polymer volume does not change during the mixing process and species interaction effects can be neglected. For the mixed state in the copolymer, it is useful to define the total thickness *d* and the volume fraction *F*_*i*_ for each polymer species as
5

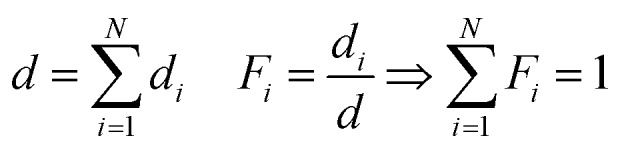




Up to now, the equations describe the most general case of a copolymer consisting of *N* species. By depositing the species *i* separately (*N* = 1), measuring its absorbance spectrum and dividing by the layer thickness measured by another technique (for example ellipsometry), the absorbance per unit length *A*_*i*_ can be directly determined. This procedure is known as direct calibration^43^ and is a specialization of the classical least squares method described in literature.^13^ The discretized version of eqn (4) for a copolymer of unknown composition can be written in vector form as

6 *A[combining right harpoon above]* = *K[combining circumflex]d[combining right harpoon above]*

where the *i*th column of matrix *K[combining circumflex]* contains the (known) homopolymer absorption spectrum *A[combining right harpoon above]*_*i*_ and *A[combining right harpoon above]* denotes the measured absorbance of the copolymer sample. This over determined system of equations can be solved for *d[combining right harpoon above]* using a nonnegative least squares method, which ensures positive (physically meaningful) *d*_*i*_ values. The total film thickness and the volume fractions are then determined by eqn (5). However, the Bouguer–Lambert–Beer approximation does not take into account electric field standing wave effects in thin layers with parallel surfaces which lead to interference fringes in the absorbance spectrum and can even alter the peak positions and their relative heights.^13^,^25^,^44^ In the case of the reference measurement of the substrate, some of the radiant power is absorbed in the sample, some is transmitted and some is reflected back to the IR source. An additional polymer layer can reduce or enhance this reflection at certain wavenumbers leading to the fringes, which can even cause negative values in the baseline-uncorrected copolymer absorbance spectrum when it is calculated according to (4), which is a shortcoming of the Bouguer–Lambert–Beer^13^ approximation. Therefore, all absorbance spectra have to be baseline corrected by subtracting the baseline.^45^ If the absorption peaks in the absorbance spectrum are separated and show much more variation than the interference fringes, the baseline can be estimated by asymmetric least squares. This baseline estimation minimizes the squared deviation from the data using asymmetric weights plus the curvature of the baseline.^46^ It uses two parameters, labeled *p* and *r* from here on, to define its properties. The weight for the squared deviation of data above the baseline is *p* and the one below the baseline is 1 – *p*. The weight for the curvature is *r*. The calculation is iterative and needs in practice 8 to 10 iterations to converge.

A simple method to verify the determined *d*_*i*_ values is to compare their sum, which is the total thickness *d*, to an accurate measurement of it. From this, the relative error of the total thickness can be calculated. The relative error of the volume fractions can then be estimated as follows: assuming the same relative thickness error *c* for each species individually, it follows that also the relative error of the total thickness is the same:
7

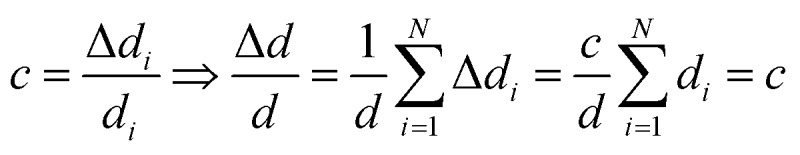




How these errors determine the error of the volume fractions can then be estimated by propagation of error of uncorrelated *d*_*i*_:
8

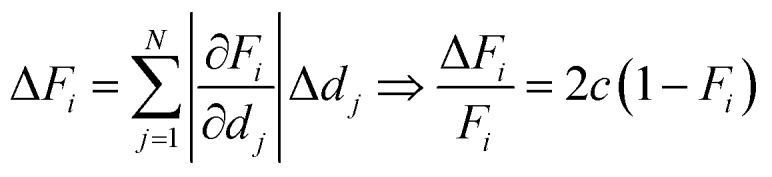




For example, the relative error of the volume fractions can be estimated by the experimentally determined error *c* for a volume fraction of 1/2.

The prerequisites for the IR-quantification method to work and the assumptions being made are the following:

• All homopolymer spectra used in the evaluation of a copolymer spectrum have linear independent absorbance spectra within their uncertainty. This is fulfilled if each homopolymer spectrum differs by at least one absorption peak from the other spectra or if a large enough peak shift is present. This requirement is necessary for the fitting process to calculate parameters with a small error.

• The total polymer volume does not depend on the spatial concentration distribution of the species and species interaction effects can be neglected. This is required so that the homopolymer spectra can be related to the copolymer spectrum *via* Bouguer–Lambert–Beer's approximation.

• The respective baselines vary much less than the absorbance spectra themselves and the absorption peaks are separated such that the baseline can be estimated by the asymmetric least squares method.

Depending on the polymerization process used for the synthesis, additional requirements can be necessary. For iCVD polymerization, the following premises have to be met in addition:

• The number of vinyl bonds that polymerize in the homopolymers is the same as in copolymers and no other bonds are formed. Otherwise more degrees of freedom for the copolymer absorbance spectrum would have to be taken into account.

• Likewise, the inclusion of radical initiators is the same during homopolymerization and copolymerization.

## Experimental

### Materials

The monomers 2-hydroxyethyl methacrylate (HEMA, 97%), methacrylic acid (MAA, 99%) and 1*H*,1*H*,2*H*,2*H*-perfluorodecyl acrylate (PFDA, 97%) as well as the cross-linker ethylene glycol dimethacrylate (EGDMA, 98%) and the initiator *tert*-butyl peroxide (TBPO, 98%) were obtained from Sigma-Aldrich (Germany) and used without further purification. As substrates, single side polished silicon wafers with a native oxide (1.7 nm) were used (Siegert Wafers, Germany).

### Initiated chemical vapor deposition

Three different copolymer types were deposited by iCVD in various compositions:

• p-(MAA–EGDMA) (32 samples)

• p-(MAA–EGDMA–HEMA) (15 samples)

• p-(PFDA–EGDMA) (4 samples)

The monomers were heated to ease vaporization (MAA to 70 °C, HEMA to 75 °C and EGDMA and PFDA to 80 °C) and were fed to the processing chamber through a heated mixing pipe. Needle valves allowed control on the individual flowrates, which were varied for MAA between 2.4 and 4.4 sccm, for EGDMA between 0.00 and 0.34 sccm, for HEMA between 0 and 0.8 sccm and for PFDA between 0.15 and 0.20 sccm to achieve polymers in different compositions. The initiator TBPO was flown into the chamber at room temperature *via* a mass flow controller. The working pressure was controlled by a butterfly valve connected to a roughening pump, and was varied between 350 and 800 mTorr, depending on the copolymer to be deposited. The substrate temperature was fixed to values between 25 and 30 °C by a heater/chiller. A more detailed description of iCVD setups in general and the experimental procedure can be found in literature.^47^

### Characterization

The thicknesses of the polymer layers were measured using a commercial spectroscopic ellipsometer (M-2000V, J.A. Woollam Co. Inc., USA). For each sample, spectral data in the wavelength range from 370 to 1000 nm were collected at three different incidence angles (65, 70 and 75°). The experimental data were fitted using the Cauchy model since the polymers are transparent in the measured wavelength range. The resulting thicknesses are consistent within about ±1% which was observed from multiple measurements of the same sample at different positions.

Absorbance spectra of all samples were collected in transmission mode on a Bruker IFS 66 v/s Fourier transform infrared spectrometer. The data were recorded in the wavenumber range 400–4000 cm^–1^ at a resolution of 4 cm^–1^ and a zero filling factor of 8. A triangular apodization function was used. The baseline estimation was performed using *r* = 1 × 10^8^ and *p* = 5 × 10^–3^ for all samples. These values were empirically determined by visually comparing the baselines and the spectra.

X-ray photoelectron spectroscopy (XPS) spectra were recorded using a Thermo Scientific instrument equipped with a monochromatic Al-Kα X-ray source (1486.6 eV). High resolution scans were acquired at a pass energy of 50 eV and a step size (resolution) of 0.1 eV. Survey scans were acquired with a pass energy of 100 eV and a step size of 1.0 eV. Photo-electrons were collected using a take-off angle of 90° relative to the sample surface. Charge compensation was performed with an argon flood gun. All analyses were performed at room temperature. XPS measurements were performed on all homopolymers and on one sample of each different type of copolymer. For each of these samples, two different spots were analysed. The atom fractions calculated from the XPS survey scans of one spot were compared to the other and showed a mean absolute deviation of 0.0019. The average between the two spots was calculated subsequently.

## Results and discussion

### Baseline correction

**Fig. 2:**
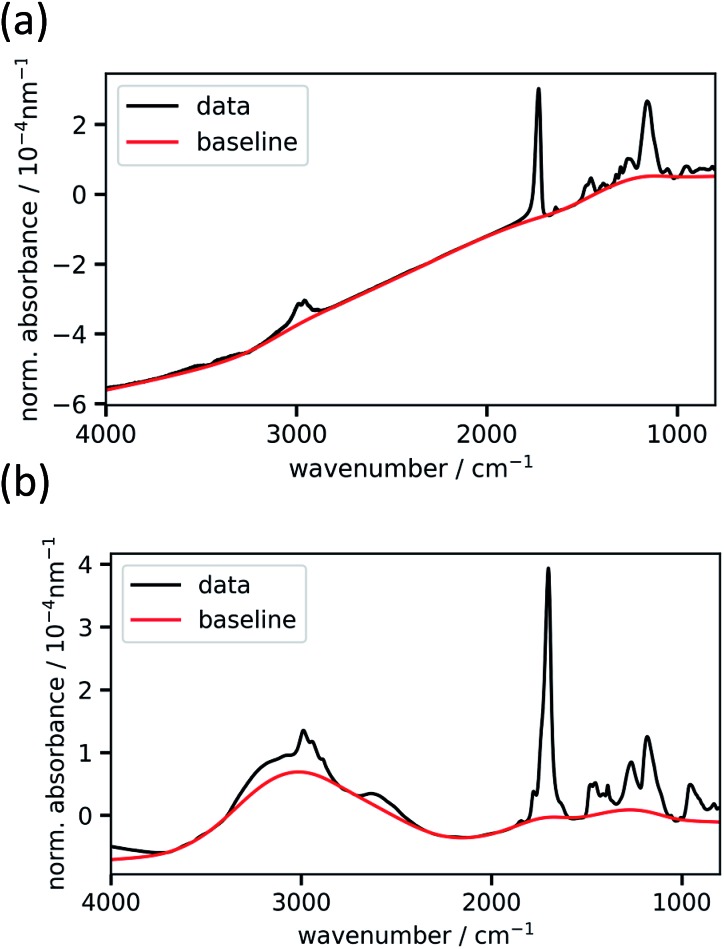
Experimental baseline-uncorrected absorbance spectra normalized by thickness of the homopolymers p-EGDMA (a) and p-MAA (b). The baseline, as estimated from asymmetric least squares (described in the text), is shown in red.

The baseline-uncorrected absorbance spectra of two exemplary homopolymer layers (p-EGDMA and p-MAA), normalized by the respective polymer thickness, are shown in Fig. 2 together with the baselines estimated by asymmetric least squares smoothing. Fig. 2a refers to a 292 nm thick film, while Fig. 2b depicts a thicker film (2020 nm). More interference fringes can be observed on the latter due to the higher thickness. This example shows the robustness of the baseline estimation algorithm, since it uses the same two parameters and still estimates the strongly different baselines to an optically plausible degree. As the homopolymer spectra serve as references for the copolymer evaluation process, thick films are desired so that the signal-to-noise ratio is maximized.

### Calculation of volume fractions

For fitting the infrared spectra, the wavenumber range from 1000 to 2000 cm^–1^ was used since it includes the strongest absorption peaks. It also excludes the range around 3000 cm^–1^ which is unstable in our setup due to fluctuations in water adsorption at the liquid nitrogen cooled detector.

**Fig. 3:**
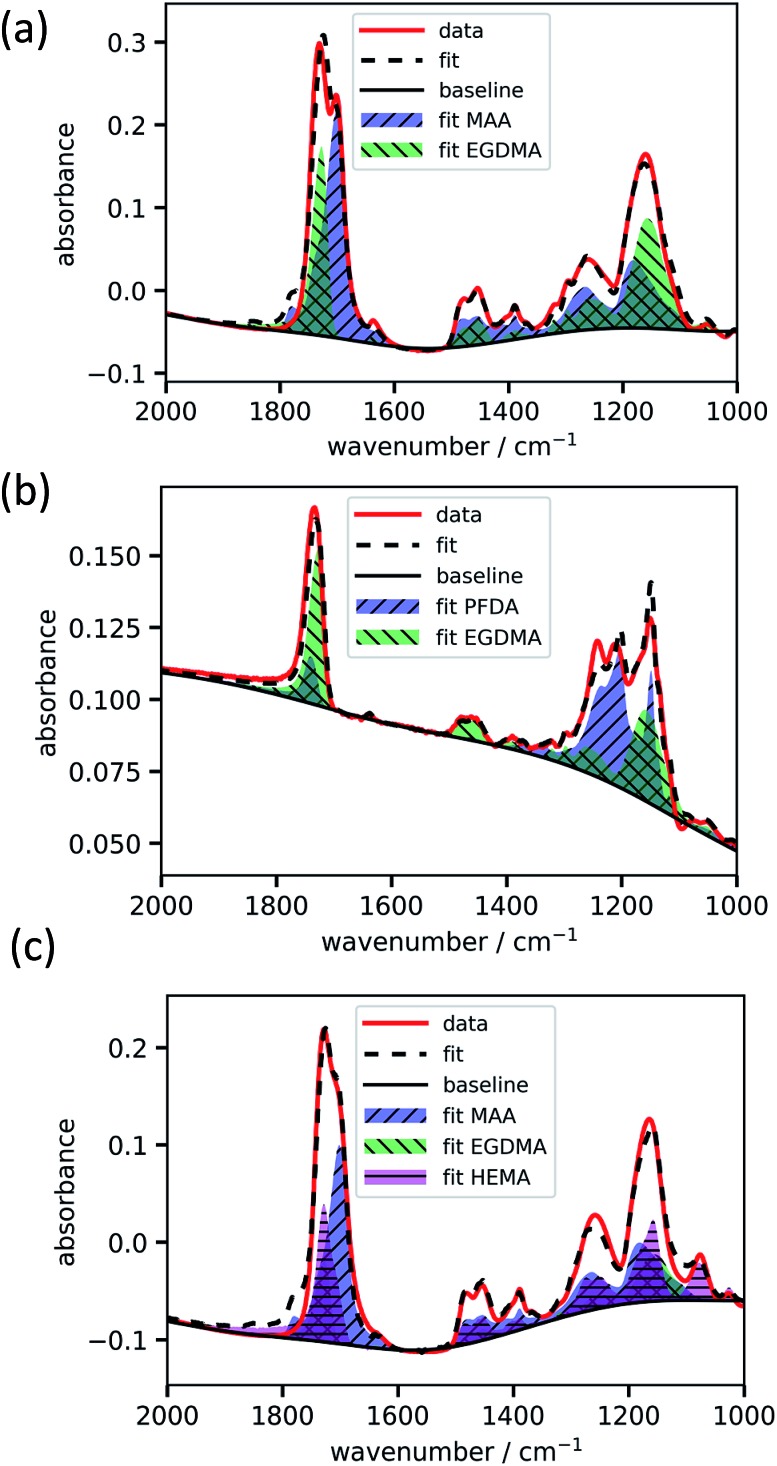
Exemplary fittings of copolymer absorbance spectra. p-MAA–EGDMA (a), p-PFDA–EGDMA (b) and p-MAA–EGDMA–HEMA (c).

**Fig. 4:**
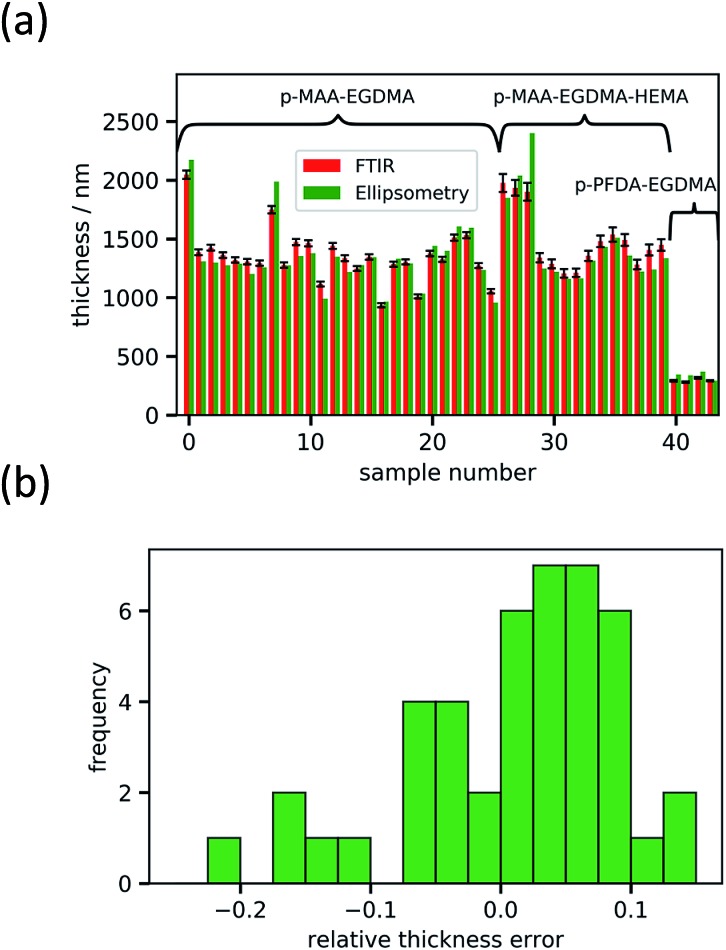
Thickness value comparison for all samples measured with FTIR and ellipsometry. The estimated error from the FTIR fitting method is shown, whereas the error of the ellipsometry measurements of about 1% is not shown (a). A histogram of the relative deviation of the FTIR value from the ellipsometry value is shown in (b).

The absorbance spectrum of two exemplary copolymers (p-MAA–EGDMA and p-PFDA–EGDMA) each consisting of two monomers as well as their baseline and fitted homopolymer spectra are shown in Fig. 3a and b, respectively. The red solid line shows the absorbance of the copolymer and the black solid line shows its baseline. The blue and green areas represent the homopolymer spectra, which span between the baseline of the copolymer spectrum and the sum of the same and the baseline corrected homopolymer absorbance spectrum. The dashed line is the fit calculated by minimizing the squared differences between the copolymer data and the linear combination with positive coefficients of the homopolymer absorbance spectra, all baseline corrected. The fits overlap very well with the experimental spectra and allow an estimate of the EGDMA volume fraction of 0.47 ± 0.02 in case (a) and 0.22 ± 0.02 in case (b). The peaks which distinguish MAA and EGDMA (Fig. 3a) the best are the C=O peaks at 1730 cm^–1^ (EGDMA) and at 1703 cm^–1^ (MAA). For PFDA and EGDMA, the components can be discriminated by their peaks around 1200 cm^–1^ (Fig. 3b). The total thicknesses estimated by the fit are 1306 ± 23 nm for case (a) and 317 ± 7 nm for case (b), while ellipsometric measurements yielded thicknesses of 1202 nm and 371 nm. An absorbance spectrum for a copolymer consisting of MAA, EGDMA and HEMA is shown in Fig. 3c. The additional species HEMA has a characteristic C–O peak at 1075 cm^–1^, which does not appear in the other spectra. Also in this case the model succeeded in reproducing the measured spectra, yielding a MAA volume fraction of 0.40 ± 0.02, an EGDMA volume fraction of 0.17 ± 0.02 and a total thickness of 1282 ± 41 nm. The ellipsometric measurement yielded a total thickness of 1222 nm. To further prove the method, the fitting routine was applied to more than 40 samples, yielding their respective volume fractions and the total film thicknesses. The total thicknesses were compared to the thicknesses measured by ellipsometry as shown in Fig. 4a. Their consistency validates the method, at least in the used parameter ranges. A histogram of the relative deviation of the thickness measured by FTIR compared to the thickness measured by ellipsometry is shown in Fig. 4b. The mean of the relative thickness errors, calculated by using eqn (9), is 0.01 and the standard deviation 0.08. The error might be for large parts due to different water uptake during the FTIR or ellipsometry measurements, leading to a different thickness of the layers as well as due to the application of the Bouguer–Lambert–Beer approximation.
9

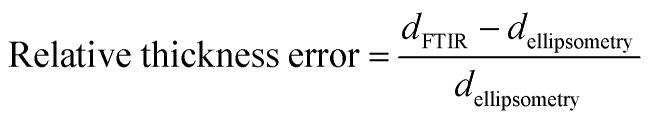




If the validity of the Bouguer–Lambert–Beer relation would not hold, *e.g. via* not negligible interaction effects, a more sophisticated calibration method could be used for which the volume fractions of copolymer calibration samples would have to be measured by another technique.^48^

To further validate the IR-quantification method, X-ray photoelectron spectroscopy (XPS) measurements were performed on the homopolymer samples as well as on one sample of each type of copolymer. The atom fractions of the homopolymers calculated from survey scans matched well with the expected atom fractions from the known chemical composition of the homopolymers with an average absolute deviation of 0.017. The measured concentration of carbon was systematically higher than expected, which could be due to surface contamination coming from the exposure of the samples to air. The high resolution XPS scans of C 1s was used to distinguish EGDMA and MAA, due to the binding energy of the component at 286.6 eV, which can be attributed to a bonding environment like –O–C*H_2_–CH_2_–,^49^ as present in EGDMA but not in MAA. The molecule number fraction of EGDMA was calculated to be 0.37 ± 0.06 leading to a volume fraction of 0.57 ± 0.09 in agreement with the 0.51 ± 0.02 calculated from the FTIR measurements. The molecule number fraction of p-MAA–EGDMA–HEMA could not be distinguished meaningfully from the survey scans because of the similar C to O ratio of the comonomers leading to a big error. For p-PFDA–EGDMA, the appearance of F in only one comonomer allowed to calculate a molecule number fraction for PFDA of 0.55 ± 0.01, which gives an estimated volume fraction for PFDA of 0.67 using its mass density and molecular weight. This value is substantially higher than the one measured by FTIR (0.47 ± 0.02). This effect has been explained by surface segregation of the fluorinated groups (only present in this copolymer) at the interface polymer–air to minimize the surface energy, as shown in literature.^50^ Due to the surface sensitivity of XPS, it yields a higher PFDA volume fraction compared to the bulk-averaged fraction measured with FTIR. To show that the IR-quantification method is not restricted to iCVD films, an exemplary blend of polymers as well as the respective homopolymers was spin coated and investigated (data not shown). The used polymer solutions were poly(3-hexylthiophene) and polystyrene in chlorobenzene. The IR-quantification program was able to properly fit the spectrum of the blend with the homopolymer spectra.

## Conclusions

An IR-quantification method was presented to calculate the volume fractions of different compositions within a copolymer thin film layer by infrared spectroscopy using automatically baseline corrected spectra in combination with the Bouguer–Lambert–Beer approximation. This method was put to test in the analysis of more than forty different copolymer samples synthesized by iCVD, a vapor-based radical polymerization technique. The total thicknesses were also calculated from the model and compared to a more accurate thickness measurement for all samples. The values were within a standard deviation as low as 8%, which is a validation of the estimates obtained with this method. Additionally, XPS measurements of an exemplary p-MAA–EGDMA sample agreed with the data gained by the IR-quantification. For p-PFDA–EGDMA, XPS data could not be used to validate the IR-quantification due to the surface sensitivity of XPS and the difference in bulk and surface composition of this copolymer. The most important requirements for the IR-quantification to work is to have homopolymers which all have linear independent absorption spectra within their uncertainty. Also, the interaction of the components has to be negligible.

The presented IR-quantification is a simple and easy to apply method for a first assessment of copolymer chemical composition in terms of volume fraction, but it can also be used to quantify blends of polymers. It minimizes analysis uncertainties and difficulties often encountered in the manual evaluation of infrared spectra and also enables the analysis of copolymers for cases, in which involved monomers possess similar chemistry. This method should be applicable not only to randomly or regularly alternating copolymers but also to block copolymers, blends of polymers and polymers synthesized by other techniques, given that the discussed prerequisites can be fulfilled.

## Conflicts of interest

There are no conflicts to declare.
